# Ginsenoside Drug Nanocomposites Prepared by the Aerosol Solvent Extraction System for Enhancing Drug Solubility and Stability

**DOI:** 10.3390/pharmaceutics10030095

**Published:** 2018-07-18

**Authors:** Cheng Tao, Jianjun Zhang, Jiexin Wang, Yuan Le

**Affiliations:** College of Chemical Engineering, Beijing University of Chemical Technology, Beijing 100029, China; 2016200052@mail.buct.edu.cn (C.T.); wangjx@mail.buct.edu.cn (J.W.)

**Keywords:** ginsenoside, drug nanocomposites, aerosol solvent extraction system (ASES), dissolution rate, anticancer activity

## Abstract

Ginsenosides are the pharmacologically active constituents of ginseng. So far, more than 30 ginsenosides have been identified and widely used in pharmaceutical formulations. However, the therapeutic applications of ginsenosides are hampered by their poor solubility and low bioavailability. In this study, we selected two of the most important ginsenosides-Re and Rh_2_ as model drugs to prepare ginsenoside drug nanocomposites (NanoGS) using the simple aerosol solvent extraction system (ASES) technique to address the poor solubility and bioavailability of these compounds. Compared with raw ginsenosides, NanoGS exhibited significantly enhanced dissolution rate owing to their low crystallinity and high surface area. Furthermore, in vitro cellular investigations showed that NanoGS-Rh_2_ exhibited outstanding anticancer activity against MCF-7 cancer cells. Therefore, this study is expected to provide a promising strategy that could optimize and broaden the applications of ginsenosides, as well as other water-insoluble drugs in pharmaceutical formulations.

## 1. Introduction

Ginsenosides are triterpene saponins that are widely used as active ingredients in various pharmaceutical formulations in clinic [1]. So far, more than 30 ginsenosides have been investigated for potential therapeutic activities, including neuroprotection [2,3,4], antioxidation [5,6,7], angiogenesis modulation [8], and anticancer activity [9,10,11]. A major ginsenoside, ginsenoside Re, was shown to exhibit multiple activities, such as cardioprotective [12], neuroprotective [13], antidiabetic effects [14], immune response enhancement [15], and memory and learning capability improvement [16]. Another remarkable member of the ginsenoside family is ginsenoside Rh_2_, an anticancer drug, which could effectively induce cancer cell apoptosis, inhibit cancer cell proliferation, and restrain tumor invasion and metastasis [17,18]. However, the bioavailability of both the ginsenosides Re and Rh_2_ was insufficient and far below the therapeutic level owing to their poor solubility and stability in the aqueous phase [19,20], which greatly limits their applications in the field of medicine and health food.

Over the past several decades, enormous approaches including polymorphism/morphology control, cocrystals/salts synthesis, inclusion complexes, amorphisation, and co-amorphisation have been explored for the development of high-performance drug formulations aimed at improving the solubilities and dissolution rates of poorly water soluble drugs [21,22,23,24,25]. Recently, micro/nano drug formulations have been produced via combination of micro/nanotechnology and pharmaceutical sciences, which is a most promising strategy for improving the bioavailability of water-insoluble drugs because the high surface area of micro/nano drugs can effectively enhance drug solubility and stability [26]. In addition, many studies showed that the low crystallinity of micro/nano drugs played a significant role in enhancing the dissolution rate [27,28]. However, the conventional methods for preparing micro/nano drugs, such as spray drying, liquid antisolvent precipitation, emulsion-solvent evaporation, and wet media milling are not suitable for many pharmaceutical compounds [29,30,31,32]. The spray drying technique may able to prepare micro/nano drugs with simple operation, but the heat applied to the drugs in the process can alter their properties. The liquid antisolvent precipitation and emulsion-solvent evaporation techniques may leave high levels of residual solvent in the products that may require further purification. Recently, the wet media milling approach has attracted much attention due to its outstanding advantages such as organic solvent-free, tunable high drug concentration, low excipient side effects, and continuous processing. However, its medical application is hampered by high mechanical stresses and prolonged milling time in the process, which may change drug properties.

Using CO_2_ dense gas technology as an alternative method for pharmaceutical and biopolymer processing is favorable, as it can circumvent both the use of organic solvents and the additional complex post-processing purification and separation stages [33,34]. One of the important CO_2_ dense gas techniques, the aerosol solvent extraction system (ASES) technique, has been widely used for preparation of micro/nano particles of pharmaceuticals and biopolymers [35,36,37]. In the ASES method, CO_2_ dense gas can be used as an antisolvent to precipitate the solute as micro/nanoparticles. However, inadequate operating parameters in the ASES process including reaction temperature, pressure, and solute concentration may greatly influence the quality of products, leading to drug particles with large particle size, broad size distribution, and nonuniform morphology, which could seriously limit its medical applications [38].

**Figure 1:**
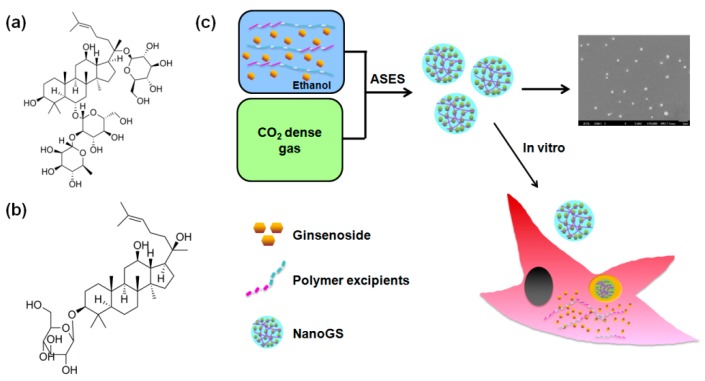
The molecular structure of ginsenoside Re (**a**) and ginsenoside Rh_2_ (**b**). (**c**) Schematic illustration showing the preparation and cellular uptake of NanoGS.

In this study, we synthesized both ginsenoside Re and Rh_2_ drug nanocomposites (denoted as NanoGS-Re and NanoGS-Rh_2_) using the ASES technique, and various operating parameters were studied to explore their effects on particle morphology, size, solubility, and stability of NanoGS. Figure 1a,b show the molecular structure of ginenoside Re and Rh_2_. The amphiphilic polymers, polyvinylpyrrolidone (PVP) and poloxamer188(POL_188_), the most popular polymer drug excipients for oral, parenteral, and topical applications, were used to prevent the aggregation and crystallization of ginsenoside particles, as well as to increase the dissolution rate and bioavailability of ginsenosides [39,40,41,42,43]. Our synthetic strategy is illustrated in Figure 1c. First, ginsenosides and polymer excipients were co-dissolved in ethanol. Subsequently, after adding the mixture solution into CO_2_ dense gas antisolvent, NanoGS particles were precipitated out. NanoGS-Rh_2_ could be taken up by cancer cells via endocytosis and further induced cell apoptosis. Finally, the in vitro anticancer activity of NanoGS-Rh_2_ was investigated against squamous cancer cells (SCC-15 cell-line) using the 3-(4,5-Dimethylthiazol-2-Yl)-2,5-Diphenyltetrazolium Bromide (MTT) and Annexin V-FITC/propidium iodide (PI) apoptosis assays.

## 2. Materials and Methods 

### 2.1. Materials

Ginsenosides Re and Rh_2_ (purity > 98%) were obtained from Aladdin Reagent Database Inc. (Shanghai, China). Polyvinylpyrrolidone (PVP) with an average M_W_ of 10,000 was provided by Beijing HengyeZhongyuan Chemical Co. Ltd. (Beijing, China). POL_188_ was obtained from Aladdin Reagent Database Inc. *N,N*-Dimethylformamide (DMF; high-performance liquid chromatography (HPLC) grade), used as a solvent, was purchased from Beijing Chemical Works (Beijing, China). CO_2_ (industrial grade), used as an antisolvent in the precipitation experiments, was obtained from Beijing RuyuanRuquan Technology Co. Ltd. (Beijing, China). All reagents and chemicals were used without further purification.

### 2.2. Synthesis of NanoGS

First, ginsenoside Re and polyvinylpyrrolidone (PVP) or ginsenoside Rh_2_ and POL_188_ at a weight ratio of 1:1 were codissolved in ethanol at a mixture concentration of 20 mg/mL. After the system reached the setting temperature and pressure, the mixture was delivered and sprayed into the precipitation vessel by using an high-performance liquid chromatography (HPLC) pump, where both the mixture solution and CO_2_ dense gas antisolvent were concurrently fed to the vessel. Once NanoGS was completely precipitated in the vessel, at least 200 mL of CO_2_ dense gas was flowed into the vessel to wash the NanoGS sample to remove the residual organic solvent. Finally, NanoGS sample was collected onto an 0.5-μm filter at the bottom of the chamber.

The drug-loading capacity (DLC) of NanoGS was determined using a UV-visible spectrophotometer (Varian Cary 50, Walnut Creek, CA, USA) using an absorption wavelength of 202 and 203 nm for NanoGS-Re and NanoGS-Rh_2_, respectively, and calculated according to the formula: DLC (wt %) = (weight of the drug/weight of drug-polymer nanocomplexes) × 100%.

### 2.3. Morphology and Particle Size of NanoGS 

The morphology of NanoGS-Re and NanoGS-Rh_2_ was examined using a model JSM-6701 scanning electron microscopy (SEM) system (JEOL, Tokyo, Japan). The particle size was determined using a Malvern Zeta Sizer Nano instrument (ZS90, Malvin City, UK).

### 2.4. Crystallinity of NanoGS

X-ray diffraction (XRD) analysis was carried out using an XRD-6000 diffractometer (Shimadzu Inc., Kyoto, Japan) to detect if there were any changes in the physical characteristics and crystallinity of the samples. Sample powder was placed in an aluminum sample holder, and the scanning speed was 5°/min from 5 to 90°.

### 2.5. Dissolution Rate of NanoGS

The dissolution rate of NanoGS in phosphate-buffered saline (PBS; 150 mM, pH 7.4) at 37 °C was measured. In each experiment, 2 mg of the ginsenoside Re or ginsenoside Rh_2_ was mixed with 3 mL of PBS, and the mixture was transferred into a dialysis bag (MWCO = 3500), which was placed in a tube containing 50 mL of PBS. Further, 3 mL of PBS was withdrawn from the tube at predetermined times, and replaced by 3 mL of fresh buffer. The cumulative dissolution rate of NanoGS was calculated according to the UV absorbance (Cary50 ultraviolet-visible spectrophotometer) at a wavelength of 202 and 203 nm for ginsenosides Re and Rh_2_, respectively.

### 2.6. In Vitro Anticancer Activity of NanoGS-Rh_2_

The cytotoxicity of NanoGS-Rh_2_ against SCC-15 cancer cells was evaluated using the 3-(4,5-Dimethylthiazol-2-Yl)-2,5-Diphenyltetrazolium Bromide (MTT) assay. Cells were plated into a 96-well plate overnight at a cell density of 3500 cells/well in 100 μL of complete Dulbecco’s modified Eagle’s medium (DMEM). Various concentrations of NanoGS-Rh_2_ were co-incubated with the cells for 48 h at 37 °C. NanoGS-Rh_2_ samples were diluted in PBS at final ginsenoside Rh_2_ concentrations of 0 to 18.0 µg/mL. Then, 20 µL of MTT stock solution (5 mg/mL) was added to the wells and incubated for another 4 h. Finally, the medium was completely removed, and 100 µL of dimethyl sulfoxide (DMSO) was added into the wells to dissolve the formazan blue crystals. The absorbance of the solution was measured using a microplate reader (Thermo Fisher, MK3, Atlanta, GA, USA) at a wavelength of 570 nm. Cell viability was determined according to the formula: Cell viability (%) = A_sample_/A_control_ × 100%, where A_sample_ and A_control_ are the absorbance values of the treated and untreated control cells, respectively. Data were expressed as the mean ± standard deviation (SD; *n* = 3).

To investigate the proapoptotic effects of NanoGS-Rh_2_ in cancer cells, an Annexin V-FITC/PI double-staining apoptosis detection assay (BD Biosciences, San Jose, CA, USA) was used. SCC-15 cells were seeded in Lab-Tek™ chambered coverglass systems (8-wells) at a density of 7000 cells/well in 200 µL of complete DMEM and cultured overnight at 37 °C in 5% CO_2_ atmosphere. Then, the cells were incubated with 50 µL of NanoGS-Rh_2_ (final ginsenoside Rh_2_ concentration = 18 µg/mL) for 48 h at 37 °C. After incubation, the cells were washed three times with PBS, stained with 5 µL of Annexin V-FITC and 10 µL of PI, incubated for another 20 min, and imaged with confocal laser scanning microscopy (CLSM) (Leica, TCS SP 5, Wetzlar, Germany).

To further quantify the in vitro apoptosis-inducing capabilities of NanoGS-Rh_2_, the SCC-15 cells were collected after incubation with NanoGS-Rh_2_ (final ginsenoside concentration = 18 µg/mL) for 48 h, and the cell density was adjusted to 1 × 10^6^ cells/mL to prepare a single cell suspension. Then, the suspended cells were stained by Annexin V-FITC and PI and detected by flow cytometry (FCM) (Beckman Coulter, MoFlo XDP, Brea, CA, USA).

### 2.7. Statistical Analysis

All statistical analyses were performed using Instat (GraphPad, San Diego, CA, USA) and SPSS 20.0 (Chicago, IL, USA). Data were statistically analyzed using one-way analysis of variance (ANOVA) to compare all pairs of data using a 95% confidence interval.

## 3. Results and Discussion

### 3.1. Preparation and Characterization of NanoGS

**Figure 2:**
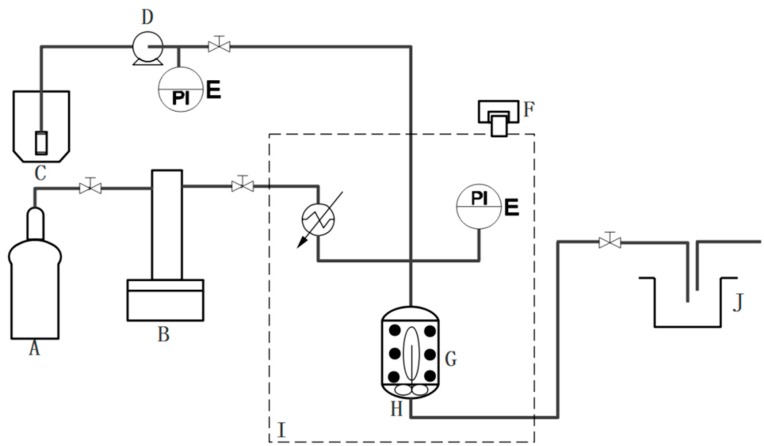
Schematic diagram of the aerosol solvent extraction system (ASES) technique: (A) CO_2_ cylinder, (B) syringe pump, (C) solution reservoir, (D) high-performance liquid chromatography (HPLC) pump, (E) pressure transducer, (F) heater, (G) precipitation vessel, (H) filter, (I) water bath, (J) solvent trap (Adapted from [44]).

**Figure 3:**
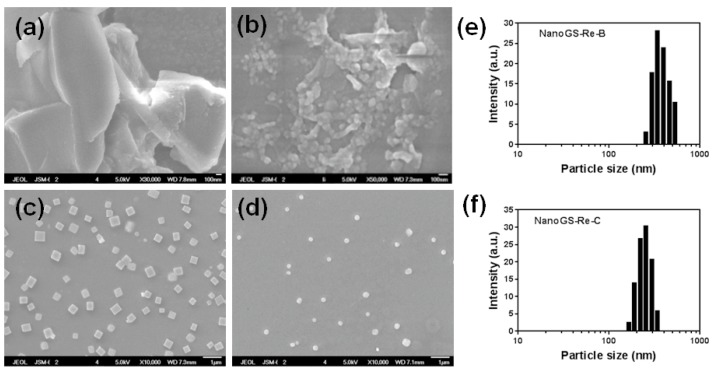
SEM images of raw ginsenoside Re (**a**), NanoGS-Re-A (**b**), NanoGS-Re-B (**c**), and NanoGS-Re-C (**d**). Dynamic light scattering (DLS) analysis of NanoGS-Re-B (**e**), and NanoGS-Re-C (**f**). The drug nanocomposites were redispersed in deionized water at a concentration of 0.2 mg/mL for all tests.

NanoGS was prepared using an aerosol solvent extraction system (ASES) (Figure 2). To explore the effects of the operating parameters of the ASES technique on NanoGS, three various operating conditions, a vapor-over-liquid (298 K and 6.6 MPa), subcritical liquid (298 K and 14 MPa), and supercritical liquid (313 K and 16 MPa) were selected for preparing NanoGS-Re-A, -B and -C, respectively. Compared with raw ginsenoside Re, which had irregular shape with a wide size distribution around several microns (Figure 3a), NanoGS-Re-A showed significantly reduced particle size; however, particle aggregation under this operating condition was significantly high (Figure 3b). As shown in Figure 3c, after increasing the operating pressure and temperature to the subcritical condition, NanoGS-Re-B exhibited regular square structure with excellent monodispersity and small particle size of approximately 300–600 nm. When the operating pressure and temperature further increased to the supercritical condition, NanoGS-Re-C exhibited fine spherical morphology, and particle size was continuously reduced to approximately 200–400 nm (Figure 3d). Figure 3e,f show the sizes of NanoGS-Re measured by dynamic light scattering (DLS). With the increase in the operating pressure and temperature, average sizes of NanoGS-Re-B and -C decreased to 342 and 255 nm, respectively, which was consistent with the results of SEM.

**Figure 4:**
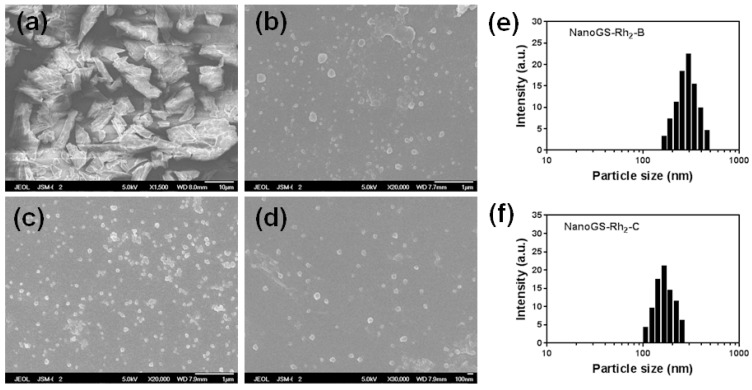
SEM images of raw ginsenoside Rh_2_ (**a**), NanoGS-Rh_2_-A (**b**), NanoGS-Rh_2_-B (**c**), and NanoGS-Rh_2_-C (**d**). DLS analysis of NanoGS-Rh_2_-B (**e**), and NanoGS-Rh_2_-C (**f**). The drug nanocomposites were redispersed in deionized water at a concentration of 0.2 mg/mL for all tests.

Furthermore, NanoGS-Rh_2_ was prepared by using the ASES method under the same operating conditions of a vapor-over-liquid, subcritical liquid, and supercritical liquid. As shown in Figure 4, similar to NanoGS-Re, with the increase in operating pressure and temperature, particle size decreased, and the average sizes of NanoGS-Rh_2_-B, and -C were 295 and 164 nm, respectively. In addition, the aggregation behavior of NanoGS-Rh_2_ was significantly improved.

Taken together, as operating pressure increased, the density of CO_2_ dense gas antisolvent in the precipitation vessel increased, which could improve the efficiency of solvent extraction. This, in turn, resulted in a significant increase in the supersaturation rate of ginsenoside solutes that might accelerate nucleation rate and restrain crystal growth, leading to the formation of NanoGS with small particle size and uniform shape [38]. Moreover, the increase in the operating temperature could further promote molecular motion and collision probability during the precipitation process, resulting in an increase in the uniformity of ginsenoside particles.

### 3.2. Crystallinity of NanoGS 

**Figure 5:**
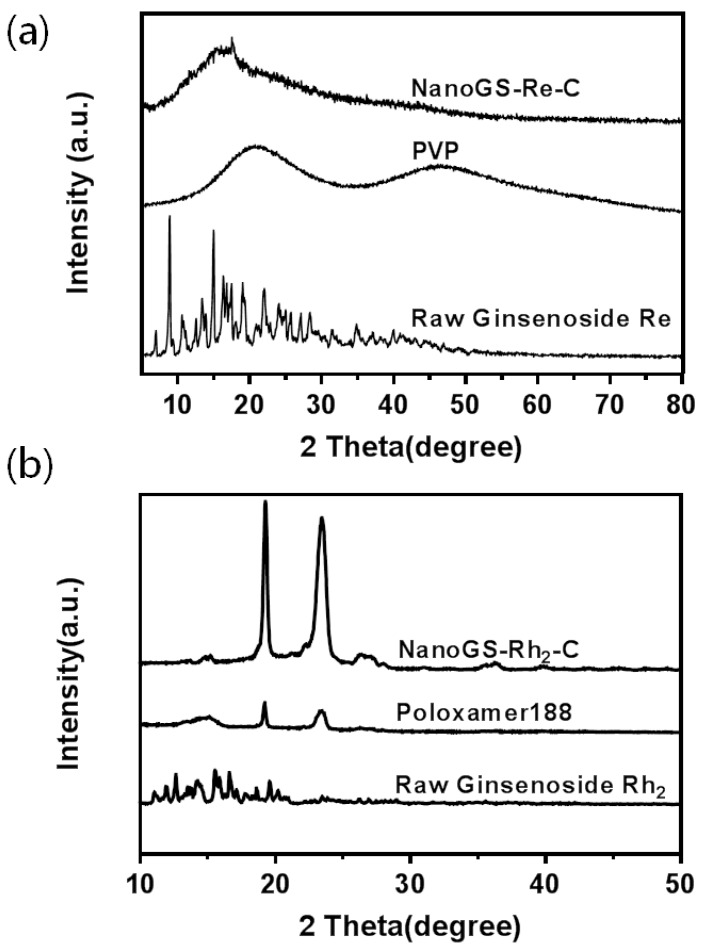
(**a**) XRD patterns of raw ginsenoside Re, polyvinylpyrrolidone (PVP), and NanoGS-Re-C. (**b**) XRD patterns of raw ginsenoside Rh_2_, POL_188_, and NanoGS-Rh_2_-C.

Reduction ofthe crystallinity of water-insoluble drugs can be beneficial to improve their dissolution profile, thereby improving the biological application of these drugs [45,46]. Figure 5a compares the XRD patterns of raw ginsenoside Re powder, PVP, and NanoGS-Re-C. Raw ginsenoside Re was highly crystalline with many sharp peaks in the XRD pattern. However, after the ASES process, NanoGS-Re showed no valuable diffraction peaks, indicating that the ASES process resulted in the formation of an amorphous state of ginsenoside Re. Similar to NanoGS-Re, NanoGS-Rh_2_ exhibited an amorphous shape in the XRD patterns (Figure 5b). In addition, XRD revealed the presence of crystalline material (approximately 5% or less) in the powder, indicating that after the ASES process, more than 90% of crystalline raw ginsenosides transformed into the amorphous state in NanoGS. This amorphous state transformation was probably attributable to the fast precipitation resulting in the formation of metastable zone of the drugs during the ASES process, which effectively decreased the crystallinity of ginsenosides Re and Rh_2_ [44]. Furthermore, previous studies proved that polymer excipients could improve the physical stability of the amorphous state of drugs by effectively inhibiting recrystallizationand grain growth of drug molecules in the coprecipitation or solvent evaporation processes owing to hydrogen-bonding interactions and the entrapment effect between polymer and drug molecules [47,48,49]. The transformation from crystalline to amorphous state during the ASES process might effectively improve the dissolution rate, as well as the bioavailability of the ginsenosides Re and Rh_2_.

### 3.3. Aqueous Stability and Dissolution Rate of NanoGS

**Figure 6:**
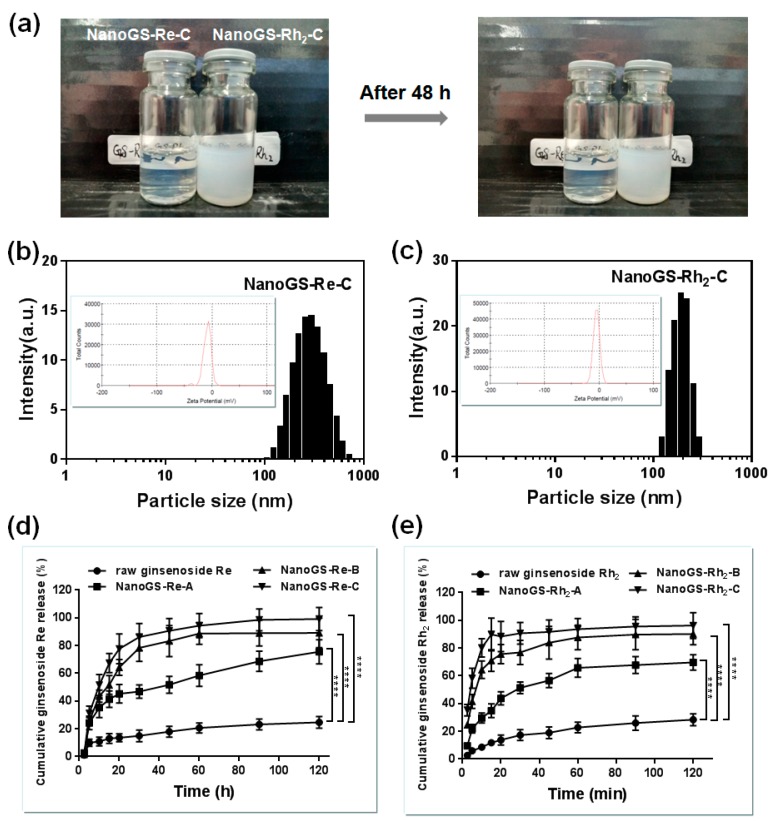
(**a**) Digital images of NanoGS-Re-C and NanoGS-Rh_2_-C dispersed in water for 48 h. DLS and zeta potential analysis of NanoGS-Re-C (**b**) and NanoGS-Rh_2_-C (**c**) after dispersing in water for 48 h. (**d**) Dissolution profiles of raw ginsenoside Re and NanoGS-Re-A, -B, and -C. (**e**) Dissolution profiles of raw ginsenoside Rh_2_ and NanoGS-Rh_2_-A, -B, and -C. Data represent the means ± SD (*n* = 3). **** p < 0.0001.

Figure 6a shows the images of NanoGS-Re and NanoGS-Rh_2_ dispersed in water. After incubation for 48h, both NanoGS-Re-C and NanoGS-Rh_2_-C retained high stability and no precipitation, suggesting that NanoGS-Re and NanoGS-Rh_2_ had excellent stability in aqueous dispersion. DLS analysis of NanoGS-Re-C and NanoGS-Rh_2_-C after dispersing in water for 48 h are shown in Figure 6b,c. Average sizes of 190 nm and 295 nm were observed for NanoGS-Re-C and NanoGS-Rh_2_-C, respectively, and there were no obvious changes compared to original NanoGS samples, proving that NanoGS possessed outstanding stability and no aggregation during the incubation period of 48 h. Furthermore, zeta potential of NanoGS-Re-C and NanoGS-Rh_2_-C were −8.49 mV and −4.79 mV, respectively, that may prove the stability of NanoGS was caused by the electrostatic repulsion among the particles.

The content of ginsenosides in NanoGS was measured using UV spectrophotometry. The drug-loading capacity (DLC) of NanoGS-Re-A, -B, and -C was 24, 31, and 38%, respectively. For NanoGS-Rh_2_, the DLC of NanoGS-Rh_2_-A, -B, and -C were 19, 23, and 32%, respectively. The results of loading efficiency further confirmed that ginsenosides were coprecipitated with the polymer excipients during the ASES process to form ginsenoside/polymer nanocomposites.

Figure 6d compares the dissolution rate of raw ginsenoside Re and NanoGS-Re. For raw ginsenoside Re, only 24.6% of ginsenoside Re dissolved during the 120 min incubation period. In contrast, the dissolution rates of NanoGS-Re-A, -B, and -C were 75.5, 89.1, and 99.1%, respectively. Figure 6e compares the dissolution rate of raw ginsenoside Rh_2_ and NanoGS-Rh_2_. Similar to NanoGS-Re, NanoGS-Rh_2_ exhibited outstanding dissolution rates, at 69.6, 90.1, and 96.2% for NanoGS-Rh_2_-A, -B, and -C, respectively. Dissolution rate results showed that the amorphous structure and high surface area of NanoGS could improve its solubility and bioavailability. Besides, the addition of water-soluble polymer excipients might further improve the dissolution performance of NanoGS [48]. Therefore, using the ASES technique with amphiphilic polymer excipients is an effective way for improving the solubility and bioavailability of water-insoluble drugs.

### 3.4. In Vitro Anticancer Activity of NanoGS-Rh_2_

**Figure 7:**
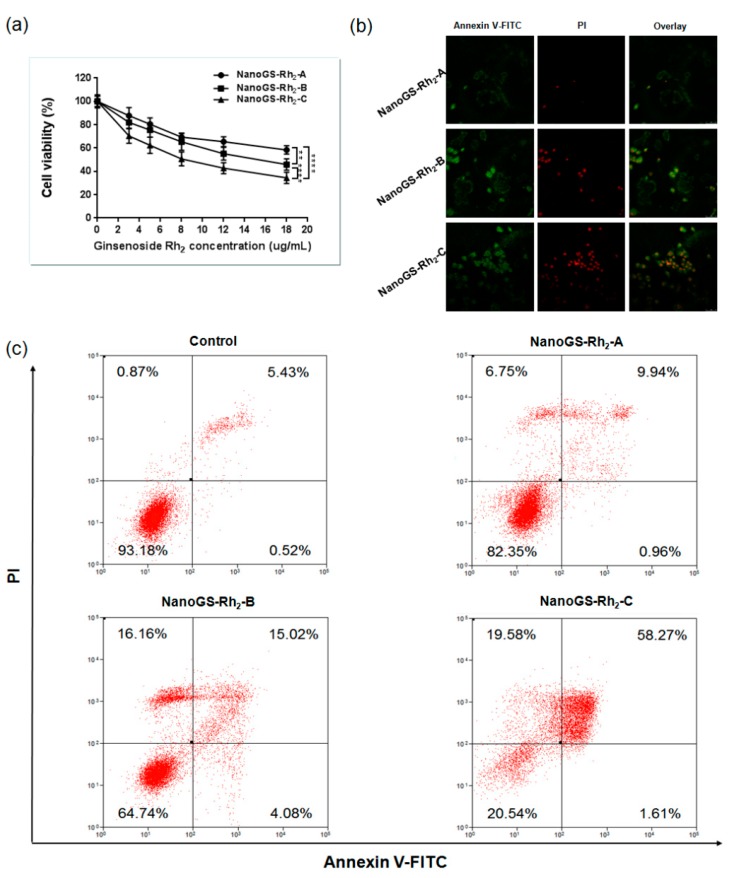
(**a**) 3-(4,5-Dimethylthiazol-2-Yl)-2,5-Diphenyltetrazolium Bromide (MTT) assay of NanoGS-Rh_2_-A, -B, and -C. Data are expressed as means with error bars from three independent tests (*n* = 3). ** p < 0.01, **** p < 0.0001. (**b**) Annexin V-FITC/PI double staining (green = early apoptotic cells, red = late apoptotic cells) of SCC-15 cells after treatment with NanoGS-Rh_2_-A, -B, and -C. Scale bar = 100 µm. (**c**) Flow cytometry (FCM) analysis of Annexin V-FITC/PI double staining of SCC-15 cells after treatment with NanoGS-Rh_2_-A, -B, and -C.

Recent studies have shown that ginsenoside Rh_2_ displayed marked anticancer activity via inhibition of cell growth and induction of apoptosis in several cancer cells [17,18]. First, we evaluated the cytotoxicity of NanoGS-Rh_2_ against SCC-15 cancer cells using the MTT assay. As shown in Figure 7a, NanoGS-Rh_2_-induced cytotoxicity was concentration-related and increased with the increase in ginsenoside Rh_2_ concentration. NanoGS-Rh_2_-A, -B, and -C at the highest ginsenoside Rh_2_ concentration (18 µg/mL) reduced cell viability to 58.3, 45.8, and 34.4%, respectively, suggesting that the decrease in particle size of NanoGS-Rh_2_ improved tumor cell death.

To evaluate the proapoptotic effects of NanoGS-Rh_2_ in SCC-15 cells, the Annexin V-FITC/PI double staining assay was used. Figure 7b shows that NanoGS-Rh_2_-C-treated cells showed more early and late apoptotic cells than NanoGS-Rh_2_-A and -B, which is in line with MTT assay results. Furthermore, NanoGS-Rh_2_-treated cells were double-stained with V-FITC/PI and quantified using flow cytometry (FCM). As shown in Figure 7c, NanoGS-Rh_2_-C significantly induced the onset of apoptosis (58.27%) when compared with apoptosis induction by NanoGS-Rh_2_-A (9.94%) and -B (15.02%). Taken together, these findings showed that the decrease in particle size of NanoGS-Rh_2_ resulted in more effective cytotoxicity, probably through the promotion of NanoGS-Rh_2_'s cellular uptake by endocytosis [50].

## 4. Conclusions

In summary, we successfully prepared ginsenoside Re and Rh_2_ drug nanocomposites using the ASES technique. Operating pressure and temperature were the main factors that influenced particle size, morphology, and monodispersity. Furthermore, NanoGS exhibited good dispersibility in aqueous phase and an extremely excellent dissolution profile in water owing to the amorphous structure, high surface area, and amphiphilic polymer excipients. Finally, the in vitro anticancer activity of NanoGS-Rh_2_ was investigated. NanoGS-Rh_2_ exhibited outstanding cytotoxicity against SCC-15 cancer cells. Moreover, we believe that this synthetic strategy might not be limited to ginsenosides, but can be applied to various poorly water-soluble drugs for therapeutic applications.
